# An electronic nose using a single graphene FET and machine learning for water, methanol, and ethanol

**DOI:** 10.1038/s41378-020-0161-3

**Published:** 2020-05-18

**Authors:** Takeshi Hayasaka, Albert Lin, Vernalyn C. Copa, Lorenzo P. Lopez, Regine A. Loberternos, Laureen Ida M. Ballesteros, Yoshihiro Kubota, Yumeng Liu, Arnel A. Salvador, Liwei Lin

**Affiliations:** 1 Berkeley Sensor and Actuator Center & Department of Mechanical Engineering, University of California at Berkeley, Berkeley, CA 94720 USA; 2 Materials Science and Engineering Program, College of Science, University of the Philippines Diliman, 1101 Quezon City, Philippines; 3 National Institute of Physics, College of Science, University of the Philippines Diliman, 1101 Quezon City, Philippines

**Keywords:** Electrical and electronic engineering, Sensors, Electronic devices, Nanosensors

## Abstract

The poor gas selectivity problem has been a long-standing issue for miniaturized chemical-resistor gas sensors. The electronic nose (e-nose) was proposed in the 1980s to tackle the selectivity issue, but it required top-down chemical functionalization processes to deposit multiple functional materials. Here, we report a novel gas-sensing scheme using a single graphene field-effect transistor (GFET) and machine learning to realize gas selectivity under particular conditions by combining the unique properties of the GFET and e-nose concept. Instead of using multiple functional materials, the gas-sensing conductivity profiles of a GFET are recorded and decoupled into four distinctive physical properties and projected onto a feature space as 4D output vectors and classified to differentiated target gases by using machine-learning analyses. Our single-GFET approach coupled with trained pattern recognition algorithms was able to classify water, methanol, and ethanol vapors with high accuracy quantitatively when they were tested individually. Furthermore, the gas-sensing patterns of methanol were qualitatively distinguished from those of water vapor in a binary mixture condition, suggesting that the proposed scheme is capable of differentiating a gas from the realistic scenario of an ambient environment with background humidity. As such, this work offers a new class of gas-sensing schemes using a single GFET without multiple functional materials toward miniaturized e-noses.

## Sensors: Graphene and machine learning sniff out gases

A sensor combined with machine learning algorithms makes an effective ‘electronic nose’ to distinguish different gases, according to research from the United States. The new approach, developed by a team led by Takeshi Hayasaka of the University of California, Berkeley, combines selectivity, low cost, and low power consumption without needing different materials to sense different gases. A graphene field effect transistor is used as a sensor, and four parameters of its conductivity profile are used as inputs to a machine learning classifier. With enough data, the classifier could identify water, methanol, and ethanol vapors. The researchers also showed that it could distinguish water and methanol in a mixture. These findings are an important step towards a miniaturized e-nose, which would be useful in areas such as environmental and safety monitoring, petrochemical processing, and other industries.

## Introduction

Miniaturized gas sensors are expected to witness a high demand in the next decade in various sectors, including industrial, consumer electronics, automotive, medical, environmental, and petrochemical fields, due to the small footprint, low power consumption, and low cost^1–3^. The major driving factors of the growing demand include continuous and real-time indoor and outdoor air quality monitoring^4,5^, increasing enforcement of occupational health and safety regulations by governments^6^, and potential consumer electronics applications^7^. By taking advantage of several unique features, miniaturized gas sensors could offer both mobile gas-sensing platforms and spatially distributed usages. These highly desirable platforms can stimulate emerging gas-sensing applications such as preventive health care and air quality monitoring with mobile devices, including smart phones. For example, some of the volatile organic compounds (VOCs) in human breath are known as biomarkers for clinical diagnostics, whereas NH_3_ and NO are related to *Helicobacter pylori* infections of the stomach and asthma, respectively^8^. The detection of VOCs such as methanol and ethanol has drawn great attention, as the former is extensively used in various industries as an important solvent and raw material^9,10^ and the latter has been studied for breath analysis and food industries^11^. On the other hand, spatially distributed gas-sensing platforms are suitable to monitor air pollution (e.g., CO, NO_2_, and SO_2_) with high spatial resolution^5^.

Different gas sensors have been demonstrated by combining key sensing principles/materials and micro/nanofabrication technologies^12,13^. Metal oxide semiconductor (MOS) gas sensors have been widely used since their emergence in the 1970s due to their high sensitivity and low cost^14,15^, and they have been further miniaturized in recent years with <10 mm^2^ or smaller footprints^15,16^; however, poor gas-sensing selectivity has been a long-standing issue^12^. In addition, the gas-sensing principle has relied on the reactive oxygen species on the MOS surface such that the sensor has to operate at high temperature (typically >200 °C)^17^ with relatively high power consumption (typically on the order of 10 mW). In contrast, optical-type gas sensors generally have high selectivity owing to the wavelength-specific gas-sensing principle. However, the relatively large size, complexity of the engineering configuration, and production cost are several limiting factors for widespread applications of optical-type gas sensors^12^.

Artificial olfactory systems (electronic noses or e-noses) have been promising tools to tackle the gas selectivity issue for MOS-based gas sensors^18–20^. The biological olfactory organ in nature has the capability for gas discrimination by using the combination of (1) cross-sensitive olfactory receptor arrays, (2) olfactory codes, and (3) the recognition system (brain). The gas selectivity is achieved by the uniqueness of the generated olfactory codes. An e-nose system may comprise similar artificial components, including (1) an array of gas sensors, (2) output vectors, and (3) a pattern recognition algorithm. The generated output vectors can be projected to an abstract space called the feature space for subsequent analyses. Although the concept of e-nose appeared in the late 1980s^21–23^ and intensive studies followed in the 1990s^24–28^, e-nose systems are not commercially successful today except for some minor usage in specialized industries^29,30^.

**Fig. 1:**
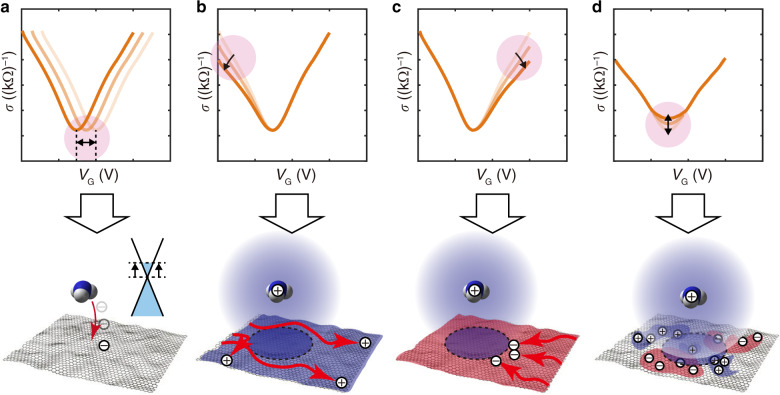
Schematic illustrations of the conductivity profiles versus the applied gate voltage and the corresponding physical phenomena over a graphene field-effect transistor (FET). **a** (Top) A gas molecule can cause the lateral movement of the conductivity profile and the movement of the charge neutral point; (bottom) the physical phenomenon of the charge transfer between a gas molecule and graphene and the carrier concentration change in the band diagram. **b** (Top) The slope in the hole branch can be altered due to the gas molecule; (bottom) the Coulomb interactions between the gas molecule and the holes. **c** (Top) The slope in the electron branch can be altered due to the gas molecule; (bottom) the Coulomb interactions between the gas molecule and the electrons. **d** (Top) The height of the charge neutral point is changed due to the gas molecule; (bottom) the modulated residual carrier concentration in the graphene

Previously, a graphene field-effect transistor (GFET) was demonstrated as a gas sensor with unique features, including ultralow power consumption (typically on the order of 10 μW) at room temperature with V-shaped conductivity profiles^31,32^; however, it suffered from poor gas-sensing selectivity^33^. Here, we propose a novel gas-sensing scheme by combining the e-nose concept and decoupled electrical signals of a single GFET to achieve selectivity, miniaturization, low cost, and low power consumption without using multiple functional materials. In the proposed scheme, the measured V-shaped conductivity profiles are decoupled into four distinctive physical properties combined with other parameters^34^:

1 $$\mu _{\mathrm{e}} = \frac{1}{c_{\mathrm{G}}}\frac{\Delta \sigma _{\mathrm{e}}}{\Delta V_{\mathrm{G}}}$$

2 $$n_{{\mathrm{e}}/{\mathrm{h}}} = \frac{c_{\mathrm{G}}}{e}|V_{{\mathrm{NP}}}|$$

3 $$\mu _{\mathrm{h}} = \frac{1}{c_{\mathrm{G}}}\frac{|\Delta \sigma _{\mathrm{h}}|}{\Delta V_{\mathrm{G}}}$$

4 $$\frac{n^ \ast}{n_{{\mathrm{imp}}}}= \frac{1}{20}\frac{h}{e^2}\sigma _0$$

where *μ*_e_ is the electron mobility; *μ*_h_ is the hole mobility; *c*_G_ is the gate capacitance per unit area; Δ*σ*_e_ is the change in electron conductivity; Δ*σ*_h_ is the change in hole conductivity; Δ*V*_G_ is the change in gate voltage; *n*_e_ is the electron concentration; *n*_h_ is the hole concentration; *e* is the elementary charge; *V*_G_ is the gate voltage; *V*_NP_ is the gate voltage at the neutrality point (NP); n* is the residual carrier concentration; *n*_imp_ is the charged impurity concentration; *h* is Planck’s constant; and *σ*_0_ is the minimum conductivity at the NP. These physical properties are influenced by the gas molecules on the surface of graphene^32,34–36^ to hold gas-specific information, such as the charge magnitude and/or dipole moment of gas molecules^35,36^. Figure 1 illustrates the measurable quantities in a conductivity profile versus gate voltage of a GFET and the corresponding physical phenomena for a graphene channel. When gas molecules approach graphene, positive or negative charge transfer can occur between the gas molecules and graphene depending on the relationship of the electron energy level, which shifts the lateral position of the NP (Fig. 1a). After the event, gas molecules can generate the Coulomb potential to cause hole–gas interactions and a modulated hole field-effect mobility to induce a slope change in the hole branch of the conductivity profile (Fig. 1b). Similarly, the electron field-effect mobility may be modulated by the attractive Coulomb force to induce a slope change in the electron branch of the conductivity profile (Fig. 1c). Near the NP (Dirac point in the electron band structure), the residual carriers and/or charged impurities can be influenced by charged gas molecules such that the ratio, n*/*n*_imp_, may be modulated to change the minimum conductivity at the NP (Fig. 1d). Therefore, it is possible to construct 4-dimensional (4D) output vectors as follows: ***q***_**1**_—the electron mobility (*μ*_e_); ***q***_**2**_—the carrier concentration (*n*); ***q***_**3**_—the hole mobility (*μ*_h_); and ***q***_**4**_—the ratio of the residual carrier concentration to the charged impurity concentration (n*/*n*_imp_). As such, the gas-specific information can be characterized within a feature space similar to that of an e-nose and resolved with pattern recognition algorithms for selective gas sensing without multiple functional materials. In fact, the four physical properties have been previously studied for gas sensing^32^ without using the 4D concept and machine-learning scheme^37^.

We experimentally investigated the 4D vectors for water, methanol, and ethanol to validate the proposed scheme under particular conditions. These particular target gases were chosen because methanol and ethanol are important VOCs, as mentioned earlier, and humidity can be a problem for GFET-based gas sensors operated at room temperature^38–41^. By using a large amount of data, our machine-learning algorithm was able to classify the 4D vectors for different gases with high consistency when they are tested individually. The gas-sensing patterns in binary mixture conditions of water and methanol vapors were qualitatively distinguished. The feasibility of identifying specific gas from the background ambient air typically mixed with various humidity levels is an important step for gas sensing toward high selectivity, miniaturization, low cost, and low power consumption.

## Results

### Measurement setup and experimental conditions

**Fig. 2:**
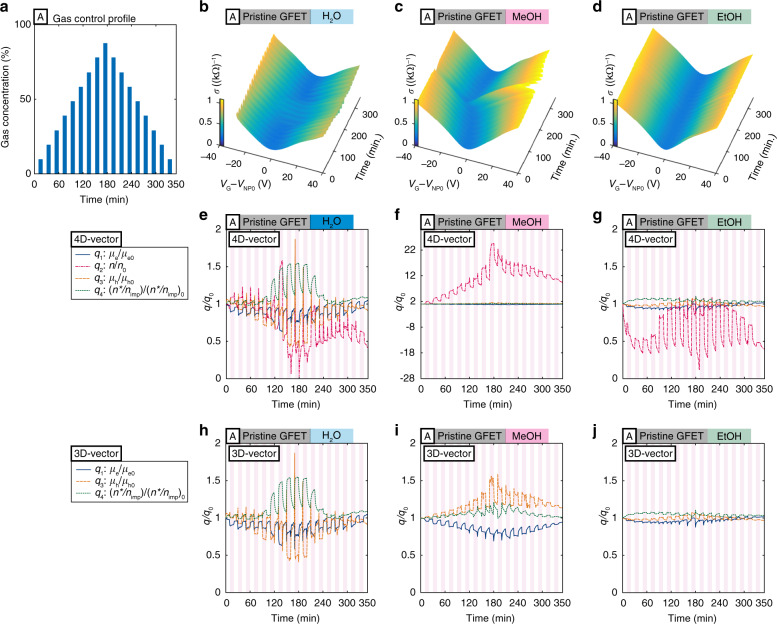
Measurement results and the converted transient 4D and 3D vectors using setup A with the pristine GFET. **a** Gas-concentration profile in test setup A. **b**–**d** Transient conductivity profiles versus the gate voltage with respect to time for water (H_2_O), methanol (MeOH), and ethanol (EtOH). **e**–**g** Relative magnitude of the converted 4D vectors versus time; **h**–**j** and relative magnitude of the 3D vectors by removing the carrier concentration vector from the 4D vectors

We prepared two different GFETs (details about the fabrication process can be found in Methods), namely, a pristine GFET and an atomic layer deposition (ALD) RuO_2_-functionalized GFET (ALD-RuO_2_-GFET), for three different experiments using three types of gases: water (H_2_O), methanol (MeOH), and ethanol (EtOH). The two different types of GFETs extend the dimension of the feature space from 4D to 8D to illustrate that the accuracy of the gas classification results can be further improved with higher dimensions. Three experimental setups, A (Fig. 2a), B (Supplementary Fig. 3a), and C (Fig. 4a), were configured to study the repeatability of the classification algorithms for individual target gases (for setups A and B) and the applicability of the scheme to binary mixtures (setup C). Throughout the study, we define the local repeatability as the repeatability within a single experimental dataset and the global repeatability as the repeatability within multiple experimental data sets. The specific gas type can be used as the variable, whereas the other parameters, e.g., the concentration and the way to produce the vapors, are set to be the same. The same measurement setup (Supplementary Fig. 2) and other common parameters were the same (Methods), such that the variables are either the tested devices and/or the gas types. We maintained the operation temperature at room temperature such that the electrical output signals are not affected by temperature variations. In the main text, we focus on the results obtained from setup A using a pristine GFET, whereas the results from all the experimental setups can be found in Supplementary Information.

### Measurement results and the converted 4D and 3D vectors

The conductivity profiles versus the gate voltage with respect to time on a pristine GFET are recorded as shown in Fig. 2b–d for H_2_O, MeOH, and EtOH, respectively. It is observed that the responses of the sensor to EtOH are small, whereas the responses to H_2_O and MeOH are relatively large and clear. These conductivity profiles were converted to 4D and 3D vectors (Fig. 2e–j) based on the proposed scheme and the relevant equations (Eqs. 1–4), and the vectors are normalized such that one can focus on the relative changes. Specifically, the 3D vectors (Fig. 2h–j) excluded the carrier concentration change in the 4D vectors in order to visualize the results in 3D feature space. Furthermore, it is useful to define the sensitivity vector, i.e., the gas-sensing pattern, ***q***_**s**_(*t*) = 100 × (***q***(t) − ***q***_**0**_)/***q***_**0**_ (%), where ***q***(*t*) is a 4D or 3D vector and ***q***_**0**_ is an initial or reference vector by using the conductivity profiles at the time right before the first gas exposure cycle starts. This definition is similar to that used in conventional gas sensors, 100 × (*R*(*t*) − *R*_0_)/*R*_0_ (%), where *R* is the resistance.

**Fig. 3:**
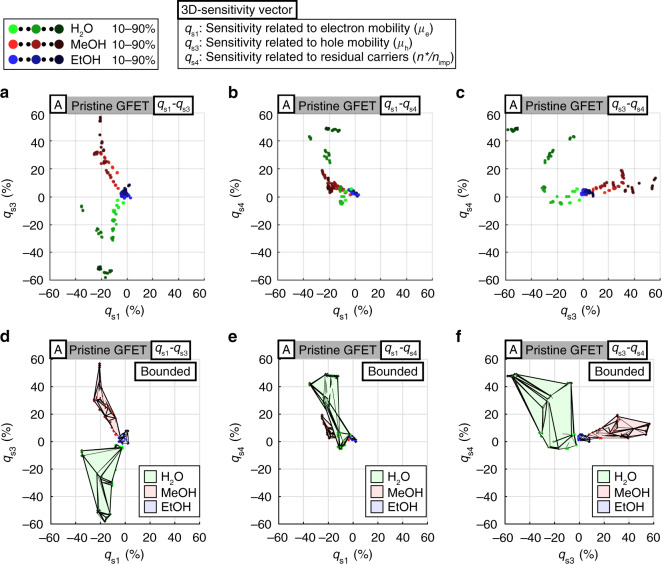
3D gas-sensing patterns projected onto 2D planes. **a**–**c** 3D gas-sensing patterns for the ascending (from 10 to 90%) cycles projected onto three representative 2D planes. **d**–**f** 3D gas-sensing patterns enclosed by triangulated boundaries for both the ascending (from 10 to 90%) and the descending (from 80 to 10%) cycles projected onto three representative 2D planes

Two different 3D gas-sensing patterns were generated and characterized as follows: (1) gas-sensing patterns representing only the ascending cycles in which the gas concentration increases from 10 to 90%; (2) gas-sensing patterns enclosed by triangulated boundaries representing both the ascending and descending (from 80 to 10%) cycles. The first pattern is utilized to examine and validate the raw data points, and the second pattern is utilized to visualize the distinctive regions for different gases. The 3D movies (Supplementary Movies 1 and 2) allow us to examine the 3D gas-sensing patterns from different angles in the 3D feature space. The representative 2D planes are shown in Fig. 3a–c for the first patterns and Fig. 3d–f for the second patterns. Figure 3a–c shows that the gas-sensing patterns have consistent trends with good local repeatability. Figure 3d–f indicates that the gas-sensing patterns are distinctive in terms of their distributions in the 3D feature space. These qualitative analyses agree with the results from experiment set B (Supplementary Fig. 3 and Supplementary Movie 3) and the results using the ALD-RuO_2_-GFET (Supplementary Figs. 5, 6, and Supplementary Movies 5–7) and thus imply the high global repeatability and validity of the proposed scheme. These results suggest that the tested gas types can be classified qualitatively by using the gas-sensing patterns.

The gas-concentration dependence on each physical property is summarized in Supplementary Fig. 8. Although most results show nearly linear relationships, some of them are nonlinear. Theoretically, the field-effect mobility should be inversely proportional to the gas concentration, whereas the carrier concentration should have linear dependency. The nonlinear behavior of the carrier concentration change, pronouncedly observed in the EtOH results, may be related to the interactions between EtOH and the pre-existing charged impurities. Despite the nonlinearity of the gas-concentration dependence, the gas-sensing patterns are qualitatively distinguishable, as they are sufficiently distinct from each other. These results suggest that the gas concentration may be better obtained by using another GFET to characterize the gas patterns in parallel, while the selectivity can be readily achieved. The gas classification capability is discussed further in a later section.

### Gas-sensing patterns of binary gas mixtures

**Fig. 4:**
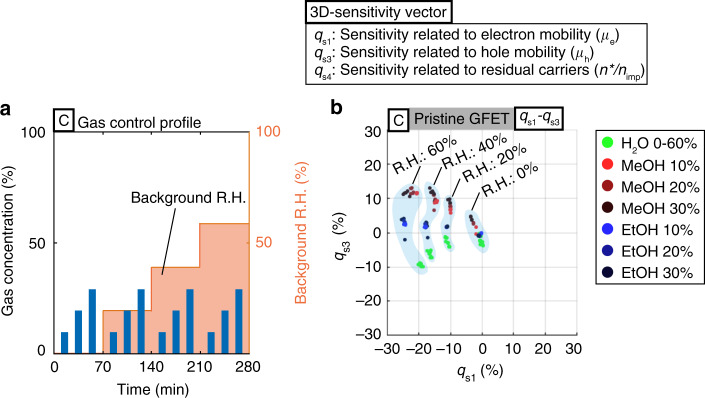
3D gas-sensing patterns of binary gas mixtures. **a** Gas-concentration profiles of the binary gas mixtures: H_2_O vapor (background humidity) and a target gas (either MeOH or EtOH). **b** Merged 3D gas-sensing patterns of the binary gas mixtures projected onto a representative 2D plot. The gas-sensing patterns are grouped by light blue colored regions, and the corresponding background R.H. levels are labeled

We are interested in distinguishing the gas-sensing patterns from those of ambient air with background humidity, as humidity can be a problem for GFET-based gas sensors operated at room temperature^38–41^. We used setup C (Fig. 4a) by varying the relative humidity (R.H.) level stepwise (red color bars), 0%, 20%, 40%, and 60%, with three purge-exposure cycles of the carrier gas, MeOH, and EtOH as the target gases (blue color bars) for each R.H. level (complete dataset in Supplementary Fig. 4). The carrier gas was used as a blank target gas, i.e., a negative control, which may only cause non-gas-related signals. Therefore, the corresponding gas-sensing patterns are considered to represent the background humidity level only. The 3D gas-sensing patterns of the three binary gas mixtures of (1) H_2_O and the carrier gas (blank), (2) H_2_O and MeOH, and (3) H_2_O and EtOH were generated for each experiment and merged into a shared 3D feature space represented by green, red and blue markers, respectively (Fig. 4b for the 2D representation and Supplementary Movie 4 for the 3D movies). In Fig. 4b, the gas-sensing patterns are grouped by light blue regions based on the corresponding background R.H. levels. To obtain the gas-sensing patterns, the reference vector, ***q***_**0**_, was defined as the vector at 10 min, which is the time right before the first gas exposure cycle starts. All ***q***(*t*) were taken from the gas exposure cycles (blue bars) such that the obtained gas-sensing patterns reflect the information of both the target gas and the background R.H. level. The results show that the gas-sensing patterns, especially for MeOH (red markers), can be distinguished visually from those with background humidity only (green markers) in Fig. 4b. In general, the gas-sensing patterns for the background humidity shift from the center to the bottom left as the R.H. level increases, whereas the gas-sensing patterns for MeOH shift to the upper side. Interestingly, the trends here qualitatively agree with the results in Fig. 3a, suggesting that the gas-sensing patterns in the binary gas mixture can be related to the superposition of the individual gas-sensing patterns tested separately. Similar trends can also be found for those using the ALD-RuO_2_-GFET (Supplementary Fig. 7 and Supplementary Movie 8).

### Classification of the gas-sensing patterns by using machine-learning analyses

A supervised machine-learning analysis was conducted to classify the gas-sensing patterns empirically. In this analysis, we examined both pristine and ALD-RuO_2_ GFETs with two setups, A and B, in which the target gases were tested individually. The goal is to distinguish three gas types, H_2_O, MeOH, and EtOH, by adopting a multiclass classification model. A multilayer perceptron classifier with a feed-forward neural network architecture was implemented and trained by using data from the two GFETs^42^. To avoid the overfitting phenomenon, which occurs when a machine-learning model undergoes too much training and may even fit to random noise such that the model fails to capture a generalized trend, a cross-validation test was performed. In general, the entire dataset was randomly shuffled in several ways and separated via a stratified split, where 20% was reserved as the testing set and the remainder constituted the training set. A stratified split ensures that each target class is adequately represented in either set. Data reserved as the testing set during each shuffle were scored by their corresponding neural network model.

**Fig. 5:**
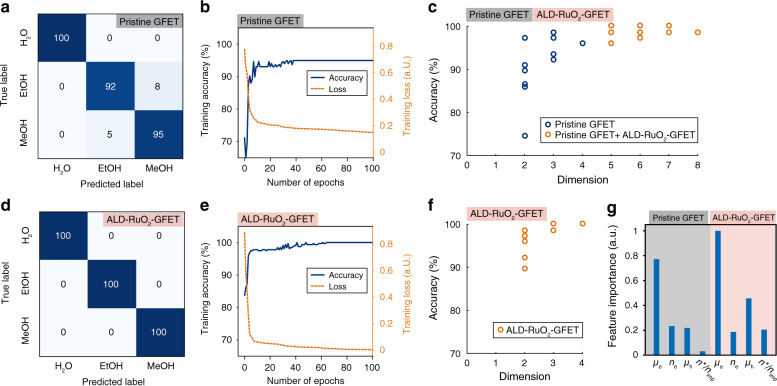
Classification of the gas-sensing patterns using the machine-learning analysis over two different experimental data sets from two different types of GFETs. **a** Confusion matrix of multiclass classification from results using the pristine GFET. **b** History of the training accuracy and the training loss from results using the pristine GFET. **c** Dimensionality dependence on the accuracy of the prediction from results using the pristine GFET for up to four dimensions, with added dimensions from results using the ALD-RuO_2_-GFET. **d**–**f** The same analysis as **a**–**c** from results using the ALD-RuO_2_-GFET. **g** Normalized feature importance with respect to the tested eight features. The four features on the left correspond to the pristine GFET and the others on the right correspond to the ALD-RuO_2_-GFET

Once the machine-learning models were trained, the confusion matrices (Fig. 5a for the pristine GFET and Fig. 5d for the ALD-RuO_2_-GFET) were used to compare the predicted labels of the testing data to their true labels. The numbers in the matrices convey the percentages of samples that were distributed among their associated label of prediction. The accuracies of the pristine GFET device and ALD-RuO_2_-GFET device were 96.2% and 100%, respectively. The cross-validation results indicated that the pristine GFET device had a mean accuracy of 95.4% and a standard deviation of 2.5%, whereas the ALD-RuO_2_-GFET device had a mean accuracy of 99.6% and a standard deviation of 0.8%. Figure 5b, e shows the accuracy and cross entropy loss history as the neural networks underwent epochs of training to minimize the loss function. A visible asymptotic state after 40 epochs implies that the model had approached convergence and that further training would not significantly improve the performance. The learning curves in Supplementary Fig. 9 compares the machine-learning models’ performance and the training set size. Both devices experienced saturation in testing accuracy as the number of training samples increases, which means that more training samples will not improve the accuracy. The narrow gap between the training and testing accuracies implies that the neural network models have low variance when exposed to unforeseen data. The ALD-RuO_2_-GFET device demonstrated a higher training accuracy than that of the pristine GFET device, which echoes their difference in classification capability mentioned above. After merely 40 epochs of training, the neural network model trained for samples measured by the ALD-RuO_2_-GFET device was able to predict 99.1% of the training data, and the time required for 40 epochs of training was 0.0519 s.

The dimensional impact on the accuracy of the model was evaluated as shown in Fig. 5c, f. For 2D and 3D models, one can choose any two out of the four features and any three out of four features for analyses, respectively. The 1D model is excluded because the scalar value cannot generate any characteristic feature. For the pristine GFET (Fig. 5c) device, different combinations of features could yield high accuracies in either 2D or 3D models compared with that of the 4D model. For the ALD-RuO_2_-GFET (Fig. 5f) results, three out of the four possible combinations in the 3D model yield 100% accuracy. By combining the features of the pristine GFET and ALD-RuO_2_-GFET devices, an 8D model can be constructed. Since the accuracy of the ALD-RuO_2_-GFET device can reach close to 100% with four features, the pristine GFET device’s 4D feature array was set as the starting point as more features from the ALD-RuO_2_-GFET device were added. As shown in Fig. 5c (red markers), adding more dimensions can result in higher accuracies than that of the 4D model. These results validate the classification capability of the multidimensional gas-sensing patterns of GFETs and suggest that an improved accuracy can be achieved by expanding the feature space to higher dimensions.

The accuracy variations in the lower-dimension (2D and 3D) models imply that some features have stronger influences on the classification study. Here, the importance of the eight features (for the two GFETs) is investigated by employing the “one-way analysis of variance (ANOVA) *F*-test” scheme, which can rank the importance of features^43^. The F-statistic is defined as the ratio of the treatment sum of squares (SST) to the sum of squares error (SSE), scaled by their respective degrees of freedom. For a feature matrix of *q* rows by *m* columns, the F-statistic is expressed as:

5 $${{F}} = \frac{{\mathop {\sum }\nolimits_{{{i}} = 1}^{{m}} \frac{{{{n}}_{{i}}\left( {{\bar{Y}}_{{i}} - \overline{\overline {{Y}}} } \right)^2}}{{{{m}} - 1}}}}{{\mathop {\sum }\nolimits_{{{i}} = 1}^{{m}} \mathop {\sum }\nolimits_{{{j}} = 1}^{{{n}}_{{i}}} \frac{{\left( {{{Y}}_{{{ij}}} - {\bar{ Y}}_{{i}}} \right)^2}}{{{{m}}({{q}} - 1)}}}}$$

where *n*_*i*_ represents the number of observations within feature *i*; $\bar Y_i$ represents the mean of feature *I;* $\overline{\overline Y}$ represents the grand mean of the entire matrix; and *Y*_*ij*_ represents the *j*th entry of feature *i*. Converting the *F*-statistic to a *p*-value by referring to the *F*-distribution, one either accepts or rejects the null hypothesis, which is that any variation observed between features is likely due to randomness. Typically, for *p*-values less than a significance level of *α* = 0.05, the null hypothesis is rejected, and the corresponding feature is considered informative. The feature with the smallest *p*-value was considered most important. Table 1 ranks the eight collective features of both sensor devices from best to worst according to the calculated *p*-values. Figure 5g qualitatively compares feature importance by taking the negative log on the *p*-value column in Table 1 and then normalizing by the most important feature. According to Table 1, all eight features had *p*-values < 0.05, which suggested that all features were in fact statistically informative to the outcome of the classification study. It is evident that the electron field-effect mobility (*μ*_e_) of both GFETs is more important than others, whereas the ratio of the residual carrier concentration to the charged impurity concentration (n*/*n*_imp_) of the pristine GFET is the least important. Therefore, the variations in the dimension dependence on the accuracy in the lower dimensions are indicative of the difference in importance between the tested features.

**Table 1:** Summary of one-way ANOVA *F*-test, ranked in descending order of feature importance.

Importance rank	Feature name	*F*-statistic	*p*-value
1	Electron mobility (*μ*_e_) of the ALD-RuO_2_-GFET	1480.34	1.08 × 10^−183^
2	Electron mobility (*μ*_e_) of the pristine GFET	831.58	6.07 × 10^−142^
3	Hole mobility (*μ*_h_) of the ALD-RuO_2_-GFET	325.78	3.78 × 10^−84^
4	Carrier concentration (*n*) of the pristine GFET	127.35	2.31 × 10^−43^
5	Hole mobility (*μ*_h_) of the pristine GFET	117.56	9.59 × 10^−41^
6	The ratio (n*/*n*_imp_) of the ALD-RuO_2_-GFET	108.68	2.69 × 10^−38^
7	Carrier concentration (*n*) of the ALD-RuO_2_-GFET	96.44	8.37 × 10^−35^
8	The ratio (n*/*n*_imp_) of the pristine GFET	13.38	2.39 × 10^−6^

## Discussion

**Fig. 6:**
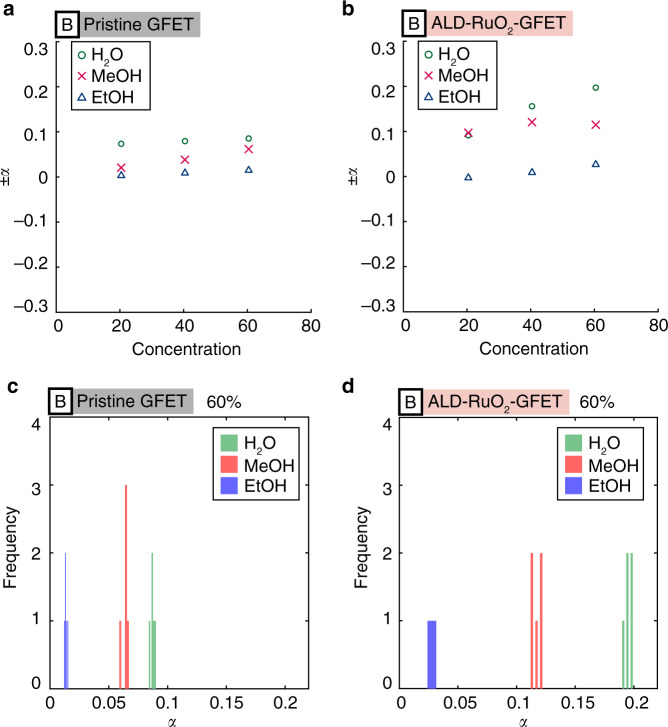
The impurity strength for H_2_O, MeOH, and EtOH. **a**, **b** The impurity strength with respect to gas concentration for the pristine GFET and the ALD-RuO_2_-GFET in setup B. **c**, **d** The histogram of the impurity strength for 60% of H_2_O, MeOH, and EtOH

Compared with other approaches using nonscalable device fabrication, special functional materials and bulky peripheral optical systems^44–47^, this work presents a practical approach to address selectivity, miniaturization, low cost and low power consumption issues at the same time. Here, we discuss the origin of the unique gas-sensing patterns. Previous studies have suggested that the electrical properties of GFETs can be dictated by the charged impurity concentration, *n*_imp_, through the following relationship (together with Eq. 4)^32,34^:

6 $$\sigma (n) = Ce\left| {\frac{n}{{n_{{\rm{imp}}}}}} \right| + {\sigma _{{\rm{res}}}}$$

7 $$\mu = \frac{C}{n_{{\mathrm{imp}}}}$$

where *C* is a constant; *e* is the elementary charge; and *σ*_res_ is the residual conductivity. The relationship of linear conductivity with respect to carrier concentration (Eq. 6) has been validated with experimental results^32^, whereas there have been some discrepancies in terms of the minimum conductivity (Eq. 4) and the field-effect mobility (Eq. 7)^32,36^. For example, inconsistent results have been observed in previous studies between the mobility and the charged impurity concentration (Eq. 7), and the possible reason has been explained as the compensation of the pre-existing charged impurities on the substrate by the incoming charged functional groups and dipolar molecules on the surface of graphene^36^. Other studies have also suggested that the dipole moment of the H_2_O molecules on graphene may have a crucial influence on the energy shift of the impurity bands with an underlying (SiO_2_) substrate^48^. With intensive studies in the last decade, it is still challenging to precisely model the impacts of gas-GFET interactions on electrical properties. Nevertheless, several measurable quantities are confirmed to be associated with gas-GFET interactions. For example, the asymmetric field-effect mobility in this study, i.e., *μ*_e_/*μ*_h_ ≠ 1 (e.g., Fig. 2e–j), can be explained by the difference in the scattering cross sections due to the attractive and repulsive Coulomb forces between the free carriers and the charged impurities, which may exist on the bottom (i.e., pre-existing charged impurities) and/or top (i.e., gas molecules) of the graphene^35,49,50^. As the Coulomb potential depends on the magnitude of the charge and/or dipole moment of gas molecules, the ratio of the carrier mobility, *μ*_e_/*μ*_h_, may provide gas-specific information. Indeed, previous studies suggest that the ratio, *μ*_e_/*μ*_h_, may be related to the impurity strength (strength of scattering due to charged impurities), *α*, via the following equation^35,49,50^:

8 $$\frac{{\mu _{\mathrm{e}}}}{{\mu _{\mathrm{h}}}} = \frac{{n_i^ + C( - \alpha ) + n_i^ - C( + \alpha )}}{{n_i^ + C( + \alpha ) + n_i^ - C( - \alpha )}},\qquad0\, <\, \alpha\, < \,\frac{1}{2}$$

where $n_i^ \pm$ represents the concentration of the positively/negatively charged impurities, and *C*(±*α*) represents the transport cross section^35,49,50^. Equation 8 allows us to estimate the impurity strength *α* in our experimental results. The numerically estimated impurity strengths are plotted in Fig. 6a, b, in which the trend of impurity strength for MeOH and EtOH are qualitatively consistent. The impurity strength of MeOH tends to increase as the gas concentration increases, while that of EtOH barely changes. Histograms for a particular condition (the gas concentration is 60% for all the gases) are provided in Fig. 6c, d from five data points for each gas, implying the uniqueness of this quantity for unique gas-sensing patterns, which qualitatively agree with the visually distinguishable gas-sensing patterns in the *q*_s1_ − *q*_s3_ plane (e.g., Fig. 3a, d) in which H_2_O and MeOH vapors can be easily distinguished. In addition, as previously suggested, the term Δ*n*_e/h_Δ*μ*_e/h_ may be related to gas-specific information^32,37^. We speculate that *q*_s4_ (~n*/*n*_imp_) reflects the interactions between the gas molecules and the pre-existing charge impurities on the substrates. As such, we attribute the origins of the unique gas-sensing patterns to the charge and/or dipole moment of the gas molecules and the interactions between the gas molecules and the pre-existing charged impurities on the substrate.

The machine-learning analyses allow us to classify the gas-sensing patterns in a systematic manner with important statistical information related to the physical properties of the tested GFETs. The testing accuracy represents a fair metric to assess the model’s ability to analyze new data, so long as the sample count representing each target gas is kept approximately equivalent to prevent a skew in prediction. The one-way ANOVA *F*-test results indicate that the electron field-effect mobility has the highest influence on the gas classification in this study. In addition, the results suggest that the importance of the features can be modulated by chemical functionalization. This information may be useful to improve the reliability of the proposed scheme further.

The potential limitations of the proposed approach are the inevitably time-consuming data collection processes and the intensive computations for the machine-learning analyses. The variations in the physical properties of GFET devices warrant a unique machine-learning model and training process for each device. From the characterization results of the prototype devices, the accuracy and cross entropy loss history (Fig. 5b, e) suggest that ~40 epochs of training are enough for a robust neural network model based on the 4D gas-sensing patterns. The total time requirement for the training process can be approximately estimated based on the number of epochs, which is almost instantaneous in this study. On the other hand, the time requirement for acquiring one piece of data during the prototype test is 1 min, which is dominated by the specifications of the peripheral measurement system and can be significantly reduced with better instruments. Another key variation is the amount of charged impurities on the substrates (boundary between graphene and SiO_2_), which can affect the charged impurity states on the substrates. Nonetheless, this issue may be alleviated by improving the quality control of the manufacturing process. Another potential issue is the influence of the various factors in the ambient environment^41^. The e-nose system based on GFET could use a temperature compensation algorithm and/or a temperature controller to eliminate the influence of temperature variations, whereas the signals from humidity could potentially be decoupled by the proposed approach. To realize an e-nose using a single GFET with the proposed scheme, other target gases should also be tested with complex backgrounds, whereas three vapors (H_2_O, MeOH, and EtOH) were evaluated with binary gas mixtures in this study.

The proposed scheme can be applied to other FET-based gas sensors, such as Si-based FETs, where the threshold voltage and the transconductance may be utilized as key parameters for multidimensional vectors. The machine-learning approach can be further extended to start with a multiclass model that distinguishes the gas mixture group, followed by a multioutput regression model of each group for the prediction of concentrations of both the target gas and common humidity values in ambient air. As long as there are sufficiently large training samples with characteristic features, the machine-learning scheme should be able to differentiate specific signatures of gas patterns and predict relevant properties. In conclusion, we have proposed and demonstrated a multidimensional gas-sensing scheme with a single GFET by utilizing distinctive 4D vectors from the results of three tested target gases and machine-learning analysis for gas classifications. As such, by decoupling the electrical signals from a single GFET, rather than adding multiple functional materials, miniaturization, low power consumption, low cost, and selectivity can be accomplished for the tested gases under particular conditions, which is a promising step toward a miniaturized e-nose.

## Materials and methods

### Fabrication and characterization of GFETs

Commercially available graphene substrates (monolayer graphene on SiO_2_/Si (300 nm/500 μm), 10 mm × 10 mm in area, Graphenea, San Sebastián, Spain) prepared by chemical vapor deposition (CVD) were used to fabricate the pristine GFETs. Metal contacts, Au/Pd (50 nm/25 nm), were patterned on the graphene substrate by a lift-off process. Subsequently, graphene channels (100 μm in width and 500 μm in length) were defined by an oxygen plasma etching process (50 W for 7–10 s). The fabricated GFETs were generally p-type (in which the majority carriers are holes); ~20 wt% polyethylenimine (PEI) solution was applied to the graphene and left for 2 h for the *n*-type counterdoping process. The PEI solution was then washed away by soaking in DI water, resulting in a charge neutral point that was shifted to close to 0 V. The fabrication process of the ALD-RuO_2_-GFET can be found elsewhere^51^. A scanning electron microscope (SEM) image of the fabricated graphene FET is shown in Supplementary Fig. 1a. The fabricated GFETs were fixed onto a ceramic package by using conductive silver paste. A typical electrical configuration of a GFET is shown in Supplementary Fig. 1b. A constant source-drain current of 100 μA is supplied between the source (S) and drain (D), and the voltage across the graphene channel is measured via two inner contacts (A) and (B) to establish the four-probe configuration. The gate voltage is applied through the Si substrate as the back gate. A conductivity profile versus gate voltage is obtained by sweeping the gate voltage from −40 to +40 V with a ramp rate of 2 V/s, as shown in Supplementary Fig. 1c. The conductivity profiles of GFETs can be decoupled into four distinctive physical properties of GFETs through Eqs. (1–4) for 4D vectors. A computed 4D vector example is shown in Supplementary Fig. 1d.

### Experimental setup for gas sensing

The gas control system consists of a dry air gas cylinder, three mass flow controllers (MFC1, MFC2, and MFC3), two vapor sources, a gas chamber, power sources, and a control and data acquisition system (Supplementary Fig. 2a). The gas concentration is determined by the ratio of two mass flow controllers, and the ratio is controlled over time based on a designated profile (e.g., Figs. 2a, 4a and Supplementary Fig. 3a) through LabVIEW (National Instruments). The gas chamber consists of a cap chamber, a GFET test chip, an IC socket, a casing, and BNC connector ports (Supplementary Fig. 2b). When the cap chamber is tightened with screws, the GFET test chip is sealed via an O-ring, and a dome-shaped space with a volume of 1 ml is formed. A schematic illustration of the cross section of the cap chamber and the GFET test chip is shown in Supplementary Fig. 2c. Throughout all the experiments, the total mass flow rate was fixed at 200 sccm such that the pressure-dependent false signal was minimized, and dry air was used as the carrier gas. The gas control profiles consist of multiple purge cycles (where only dry air is injected) and gas exposure cycles of 10 min for each test. The conductivity profiles of GFETs were acquired every minute; therefore, one gas exposure cycle contained 10 conductivity profiles. In experimental setup C, the background R.H. level was controlled by MFC1 and MFC2, and the target gas concentration was controlled by MFC3.

### Data preprocessing workflow

A supervised classification study was conducted to substantiate the selectivity of our gas sensor. The task was to train the machine-learning (ML) model for each sensor device to distinguish specific target gases with good selectivity. Data preprocessing was performed once raw data were imported to a Jupyter Notebook. During each alternation from a purge cycle to an exposure cycle and vice versa, we removed the first few samples to avoid possibly unstable data between the cycles. Afterwards, a new “label” column was created to denote the target gas species representing each sample’s feature vector. Entries in the label column were numerically coded. For a three-class study such as the three different gases tested in this work, each gas type was represented as a digit: 0, 1, or 2. The next step was to separate the entire data into a training and testing set according to an 80/20 split. The training set was reserved for the ML model to “learn” about the data and iteratively optimize the classification model, whereas the testing set was served to evaluate the algorithm’s performance by giving unforeseen data. All numeric feature values were subsequently normalized by the StandardScaler function in the Scikit-learn Python library by deducting each numeric entry by their corresponding feature’s mean and then dividing by said feature’s standard deviation^52,53^. The purpose of normalization was to prevent features that were numerically greater in value to dictate the outcome of the classification study. To prevent the distribution of the testing set from leaking into the ML model, the mean and standard deviation represented those of the training set only.

### Multilayer perceptron model

The ML model supported multiclass classification to enforce the classification of a sample to one and only one gas type. Various contemporary gas sensor applications, such as the e-nose, adopt the artificial neural network model because of its ability to model and predict complex data^26,28,42,54,55^. The multilayer perceptron (MLP) classifier, which adopts a feed-forward neural network architecture, was implemented for this study. The MLP neural network model contains three components: an input layer, an hidden layer, and an output layer. The hidden layer comprises a set of neurons, which take in a weighted linear combination of the normalized feature values from the input layer plus a bias term, and then pass through an activation function such as a rectified linear unit (RELU)^54^. The weight factor (*w*_*i,j*_) connects the *i*th entry of the input layer to the *j*th neuron of the hidden layer. Their outputs are fed to the next hidden layer(s) (should they exist) as the input until reaching the output layer, where the value of each entry correlates to the likelihood of each possible target class. The presence of the hidden layer(s) allows the neural network model to model nonlinear data, and the activation function acts as a means to buffer the noise in the data^54^. The neural network model realizes the underlying pattern in data by executing the backpropagation algorithm, which iteratively searches for the optimal weights and biases to minimize the error between the predicted label and the true label. The number of hidden layers and the number of neurons to place within each hidden layer are determined from literature research without yielding a definitive rule of thumb. However, it is ideal to keep the number of hidden layers to 2 and select the number of neurons such that the trained model does not overfit or underfit the data^54^. Scikit-learn library’s API for an MLP classifier object offers a suitably large number of hyperparameters for programmers to modulate^52,53^. The classifier object was fitted against the training set of each sensor device. Once a stopping criterion of the training process was met, the testing set was then fed to the trained classifier to evaluate the accuracy as well as other pertinent performance metrics.

### Overfitting and the cross-validation test

Machine-learning models face the problem of overfitting when a model undergoes too much training such that it fits random noise and fails to capture a generalized trend, thus producing a significant drop in testing accuracy. Although a testing dataset was explicitly put aside at the onset to evaluate the model’s robustness against new data, the concern over whether the testing set constituted a fair representation of all unforeseen likelihoods cannot be ruled out. A cross-validation test is conducted to ensure that the neural network model’s generalization performance is not too high or low by coincidence. The entire dataset was randomly shuffled several ways and separated via a stratified split, of which 20% were reserved as the testing set and the remaining constituted the training set. A stratified split ensures that each target class is adequately represented in either set. Data reserved for testing during each shuffle were scored by their corresponding neural network model. By recording the mean and standard deviation of the performance metric, such as accuracy, one can interpret whether the ML model is robust against unseen data.

## Supplementary information

Supplementary Figures

Supplementary Movie 1

Supplementary Movie 2

Supplementary Movie 3

Supplementary Movie 4

Supplementary Movie 5

Supplementary Movie 6

Supplementary Movie 7

Supplementary Movie 8
