# Supplementary material for: An electronic nose using a single graphene FET and machine learning for water, methanol, and ethanol
Source: Microsyst Nanoeng. 2020 May 18;6:50. doi: 10.1038/s41378-020-0161-3 (PMC8433337; doi:10.1038/s41378-020-0161-3)
Supplement: Supplementary file 1 — Supplementary Figures [file 41378_2020_161_MOESM1_ESM.pdf]

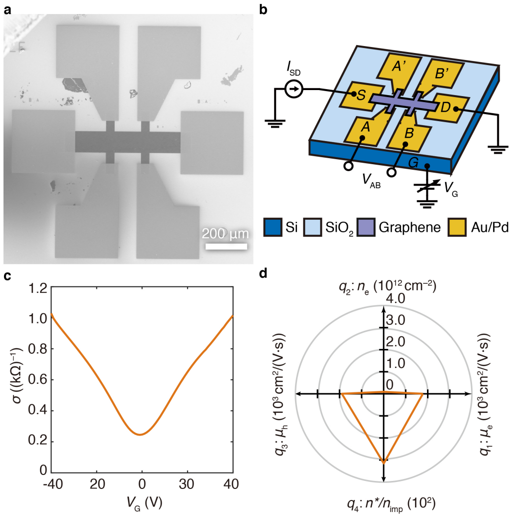

Supplementary Figure 1: Characterizations of the fabricated pristine-GFET. (**a**) Scanning electron microscope (SEM) image of the fabricated pristine-GFET. (**b**) A typical electrical configuration of a GFET: I_{SD} as a constant source-drain current, V_{AB} as the voltage across the two inner electrodes, V_G as the gate voltage. (**c**) A conductivity profile versus gate voltage of the fabricated pristine-GFET in dry air. (**d**) The converted four physical properties from the conductivity profile (**c**).

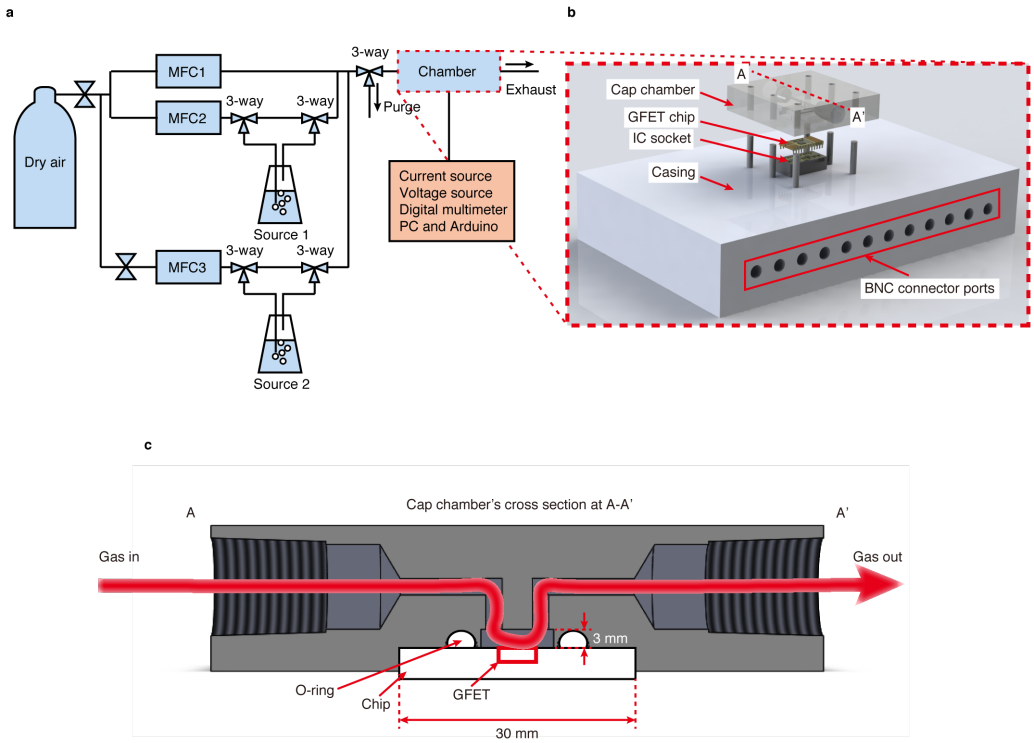

Supplementary Figure 2: Experiment setup of gas sensing. (**a**) Schematic of the gas control system comprising a dry air gas cylinder, three mass flow controller (MFC1, MFC2, and MFC3), two vapor sources, a gas chamber, power sources, and a control and data acquisition system. (**b**) Schematic of the gas chamber configuration comprising a cap chamber, a GFET test chip, an IC socket, a casing, and BNC connector ports. (**c**) Schematic of the cross sectional view of the cap chamber mounted on a GFET test chip, sealed with an O-ring.

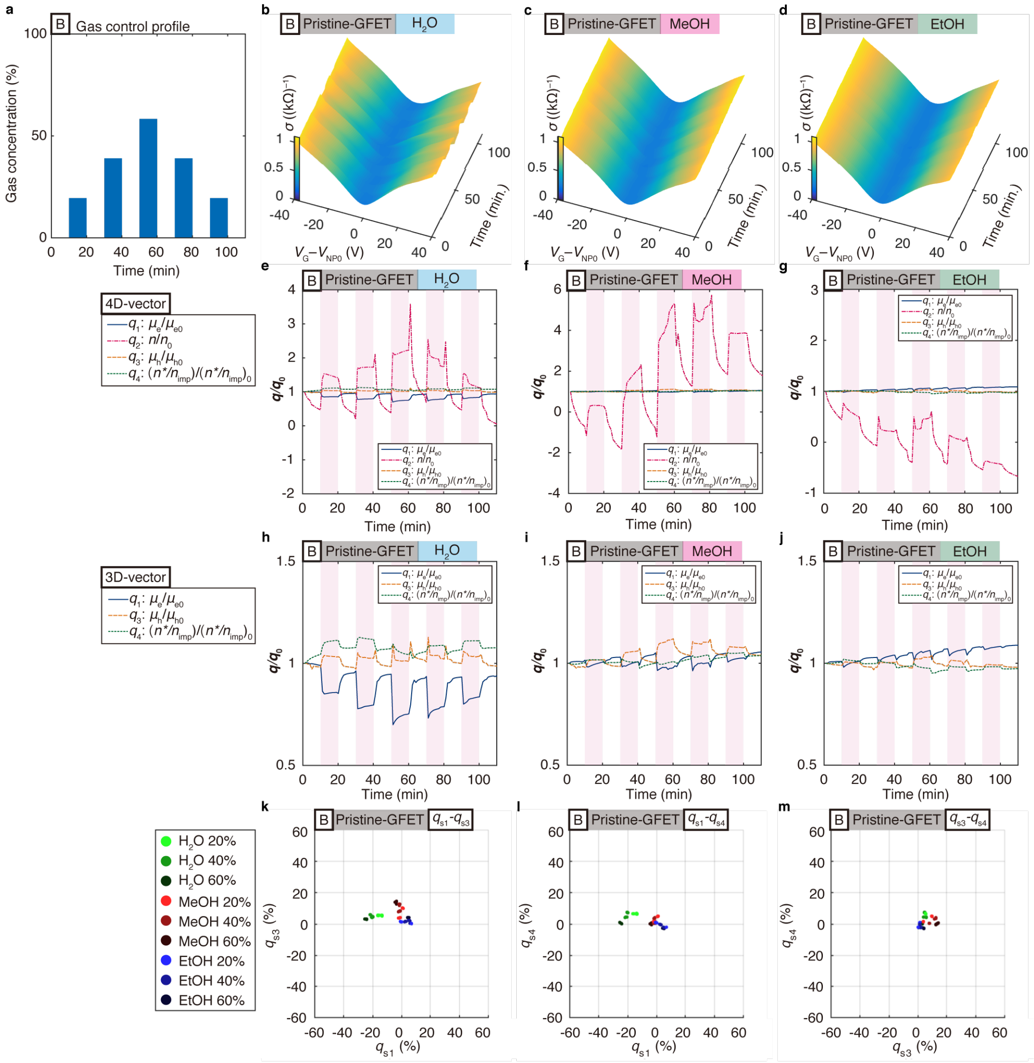

Supplementary Figure 3: Measurement results and the converted transient 4D and 3D vectors and the 3D gas sensing patterns projected onto 2D planes in experiment set B with the pristine-GFET. (**a**) Gas concentration profile of the target gases in experiment set B. (**b-d**) Transient conductivity profiles versus gate voltage with respect to time for water (H_2O), methanol (MeOH), and ethanol (EtOH) vapors. (**e-g**) Relative magnitude of converted 4D vectors versus time; (**h-j**) and relative magnitude of 3D vectors by removing the carrier concentration vector in the 4D vectors. (**k-m**) 3D gas sensing patterns for the first cycles projected onto three representative 2D planes.

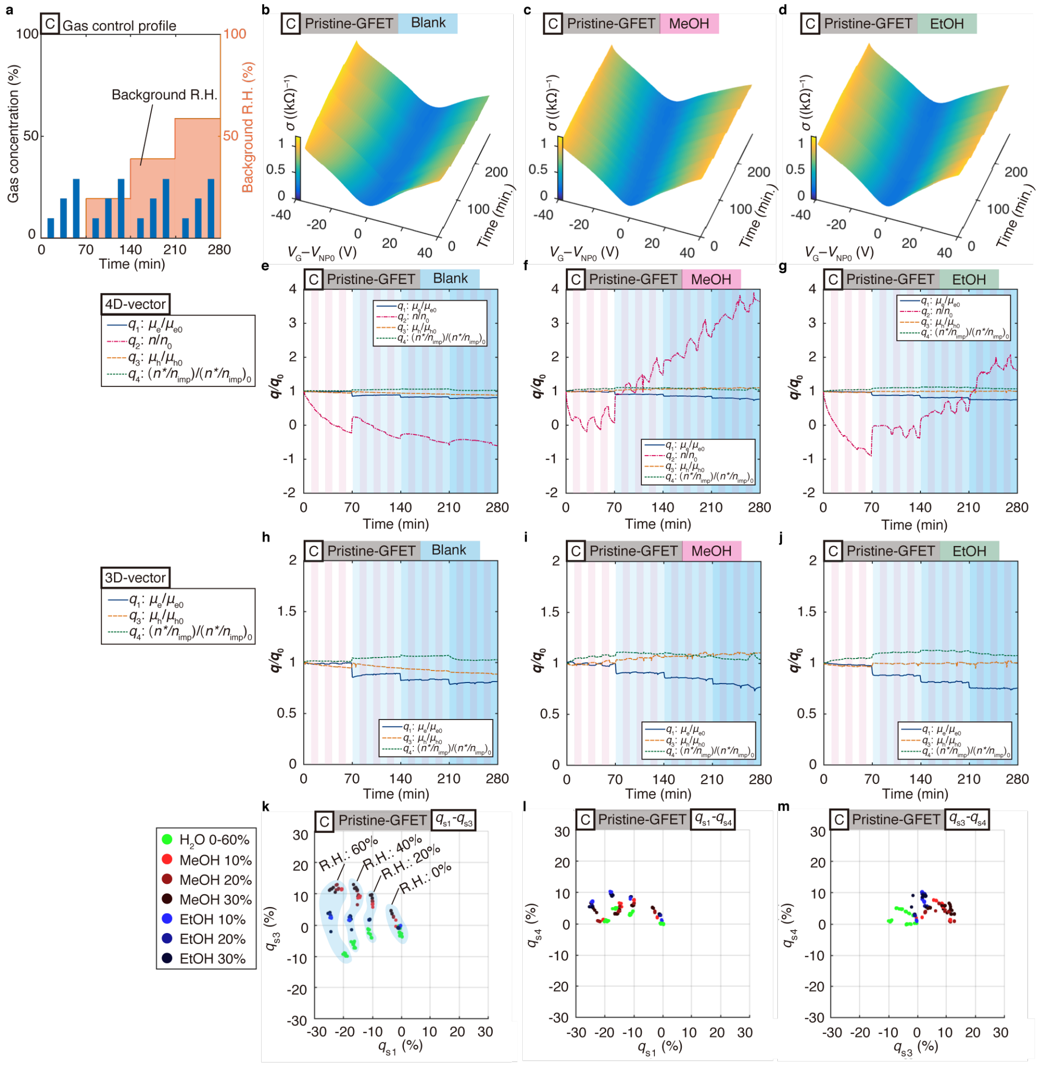

Supplementary Figure 4: Measurement results and the converted transient 4D and 3D vectors and the 3D gas sensing patterns projected onto 2D planes in experiment set C with the pristine-GFET. (**a**) Gas concentration profiles of the target gases in experiment set C. (**b-d**) Transient conductivity profiles versus gate voltage with respect to time for blank (water vapor background only), methanol (MeOH), and ethanol (EtOH) vapors. (**e-g**) Relative magnitude of converted 4D vectors versus time; (**h-j**) and relative magnitude of 3D vectors by removing the carrier concentration vector in the 4D vectors. (**k-m**) 3D gas sensing patterns projected onto three representative 2D planes.

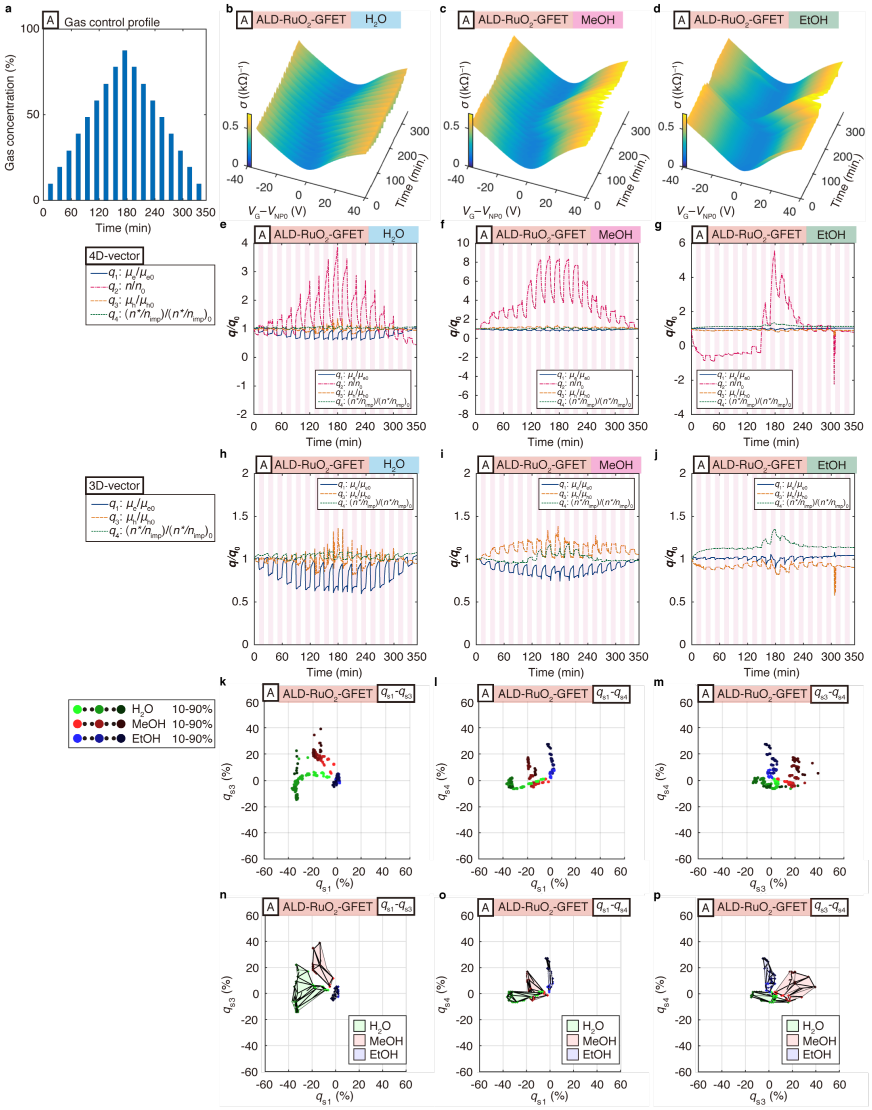

Supplementary Figure 5: Measurement results and the converted transient 4D and 3D vectors and the 3D gas sensing patterns projected onto 2D planes in experiment set A with the ALD-RuO_2-GFET. (**a**) Gas concentration profile of the target gases in experiment set A. (**b-d**) Transient conductivity profiles versus gate voltage with respect to time for water (H_2O), methanol (MeOH), and ethanol (EtOH) vapors. (**e-g**) Relative magnitude of converted 4D vectors versus time; (**h-j**) and relative magnitude of 3D vectors by removing the carrier concentration vector in the 4D vectors. (**k-m**) 3D gas sensing patterns for the first cycles projected onto three representative 2D planes. (**n-p**) 3D gas sensing patterns for the first and the second cycles with triangulated boundaries projected onto three representative 2D planes.

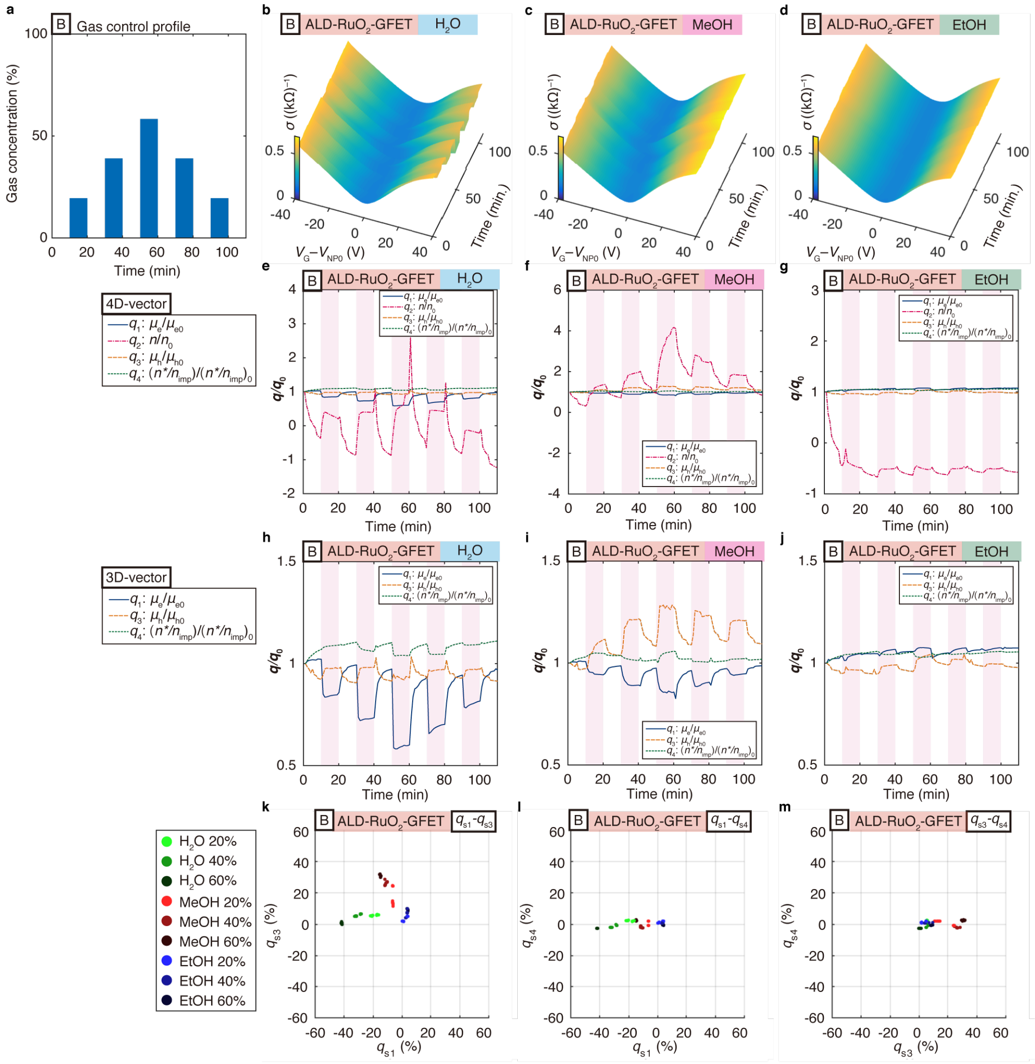

Supplementary Figure 6: Measurement results and the converted transient 4D and 3D vectors and the 3D gas sensing patterns projected onto 2D planes in experiment set B with the ALD-RuO₂-GFET. (**a**) Gas concentration profile of the target gases in experiment set B. (**b-d**) Transient conductivity profiles versus gate voltage with respect to time for water (H_2O), methanol (MeOH), and ethanol (EtOH) vapors. (**e-g**) Relative magnitude of converted 4D vectors versus time; (**h-j**) and relative magnitude of 3D vectors by removing the carrier concentration vector in the 4D vectors. (**k-m**) 3D gas sensing patterns for the first cycles projected onto three representative 2D planes.

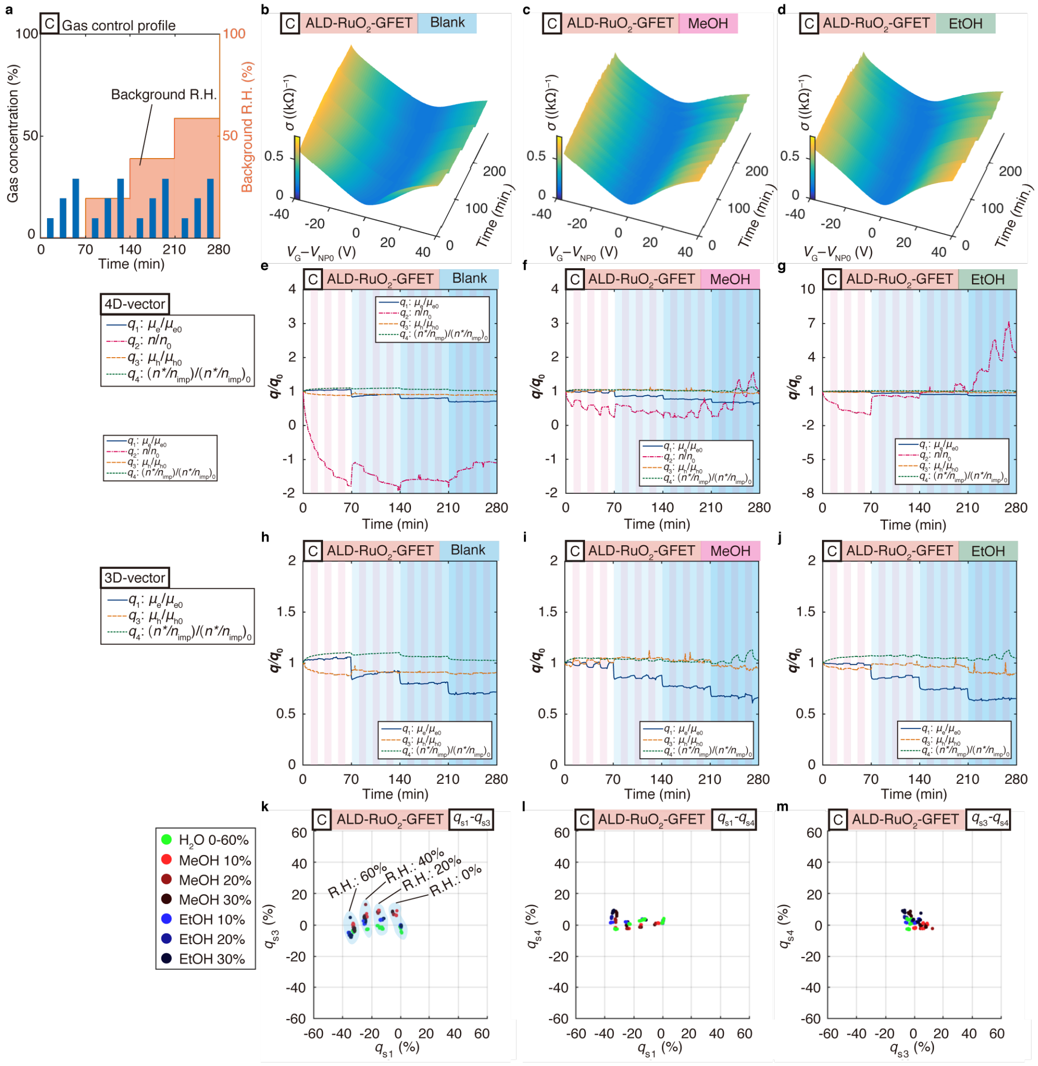

Supplementary Figure 7: Measurement results and the converted transient 4D and 3D vectors and the 3D gas sensing patterns projected onto 2D planes in experiment set C with the ALD-RuO_2-GFET. (**a**) Gas concentration profiles of the target gases in experiment set C. (**b-d**) Transient conductivity profiles versus gate voltage with respect to time for blank (water vapor background only), methanol (MeOH), and ethanol (EtOH) vapors. (**e-g**) Relative magnitude of converted 4D vectors versus time; (**h-j**) and relative magnitude of 3D vectors by removing the carrier concentration vector in the 4D vectors. (**k-m**) 3D gas sensing patterns projected onto three representative 2D planes.

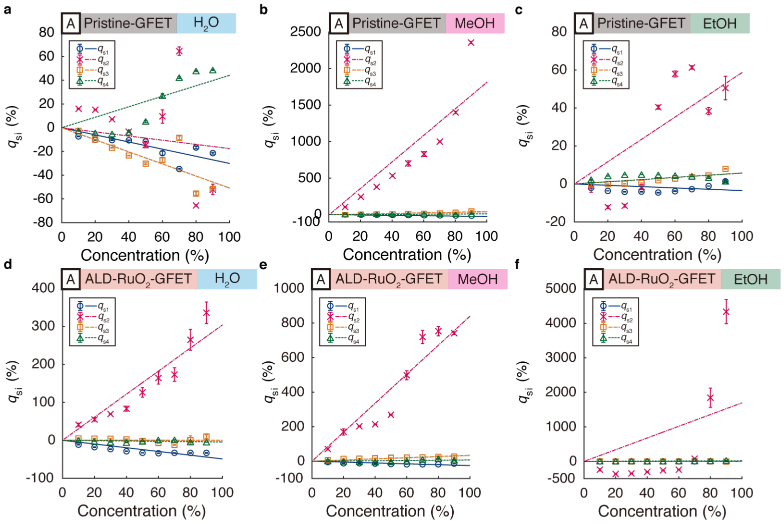

Supplementary Figure 8: Gas concentration dependence on each physical property in experiment A. (**a**-**c**) Gas concentration dependence of water (H_2O), methanol (MeOH), and ethanol (EtOH) vapors for the pristine-GFET. (**d**-**f**) Gas concentration dependence of water (H_2O), methanol (MeOH), and ethanol (EtOH) vapors for the ALD-RuO_2-GFET.

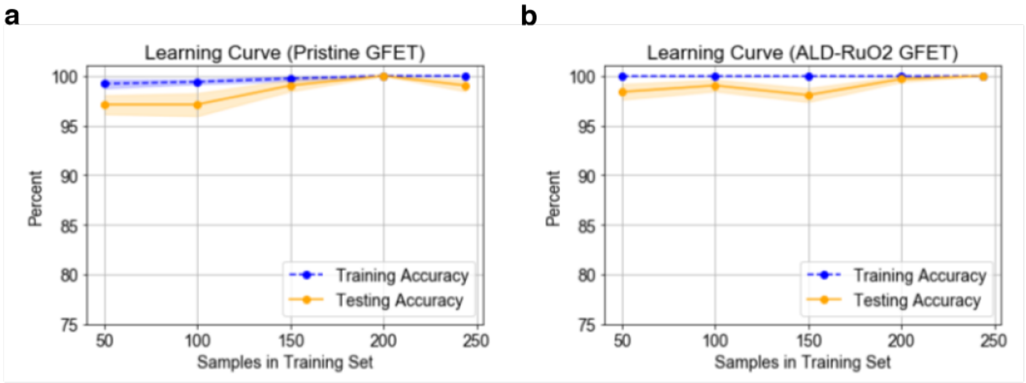

Supplementary Figure 9: Learning curve of the multi-layer perceptron classifier. (**a**) for the pristine-GFET and (**b**) for the ALD-RuO_2-GFET. The accuracies are computed by taking average of the folds' scores in a cross-validation process, and the shaded region represented margins of one standard error.

Supplementary Movie 1: 3D gas sensing patterns for the ascending (from 10% to 90%) cycles in a 3D feature space of the pristine-GFET in experiment set A. The legends are found in Fig. 3.

Supplementary Movie 2: 3D gas sensing patterns enclosed by triangulated boundaries for both the ascending (from 10% to 90%) and the descending (from 80% to 10%) cycles in a 3D feature space of the pristine-GFET in experiment set A. The legends are found in Fig. 3.

Supplementary Movie 3: 3D gas sensing patterns for the ascending (from 20% to 60%) cycles in a 3D feature space of the pristine-GFET in experiment set B. The legends are found in Supplementary Fig. 3.

Supplementary Movie 4: Merged 3D gas sensing patterns for the binary gas mixtures in a 3D feature space of the pristine-GFET in experiment set C. The legends are found in Fig. 4.

Supplementary Movie 5: 3D gas sensing patterns for the ascending (from 10% to 90%) cycles in a 3D feature space of the ALD-RuO_2-GFET in experiment set A. The legends are found in Supplementary Fig. 5.

Supplementary Movie 6: 3D gas sensing patterns enclosed by triangulated boundaries for both the ascending (from 10% to 90%) and the descending (from 80% to 10%) cycles in a 3D feature space of the ALD-RuO_2-GFET in experiment set A. The legends are found in Supplementary Fig. 5.

Supplementary Movie 7: 3D gas sensing patterns for the ascending (from 20% to 60%) cycles in a 3D feature space of the ALD-RuO_2-GFET in experiment set B. The legends are found in Supplementary Fig. 6.

Supplementary Movie 8: Merged 3D gas sensing patterns for the binary gas mixtures in a 3D feature space of the ALD-RuO_2-GFET in experiment set C. The legends are found in Supplementary Fig. 7.
